# Constructing big data prevention and control model for public health emergencies in China: A grounded theory study

**DOI:** 10.3389/fpubh.2023.1112547

**Published:** 2023-03-16

**Authors:** Huiquan Wang, Hong Ye, Lu Liu

**Affiliations:** ^1^School of Politics and Public Administration, China University of Political Science and Law, Beijing, China; ^2^School of Foreign Studies, China University of Political Science and Law, Beijing, China; ^3^School of Engineering and Technology, China University of Geosciences, Beijing, China

**Keywords:** big data, public health emergencies, epidemic prevention and control, “DSA” model, emergency management

## Abstract

Big data technology plays an important role in the prevention and control of public health emergencies such as the COVID-19 pandemic. Current studies on model construction, such as SIR infectious disease model, 4R crisis management model, etc., have put forward decision-making suggestions from different perspectives, which also provide a reference basis for the research in this paper. This paper conducts an exploratory study on the construction of a big data prevention and control model for public health emergencies by using the grounded theory, a qualitative research method, with literature, policies, and regulations as research samples, and makes a grounded analysis through three-level coding and saturation test. Main results are as follows: (1) The three elements of data layer, subject layer, and application layer play a prominent role in the digital prevention and control practice of epidemic in China and constitute the basic framework of the “DSA” model. (2) The “DSA” model integrates cross-industry, cross-region, and cross-domain epidemic data into one system framework, effectively solving the disadvantages of fragmentation caused by “information island”. (3) The “DSA” model analyzes the differences in information needs of different subjects during an outbreak and summarizes several collaborative approaches to promote resource sharing and cooperative governance. (4) The “DSA” model analyzes the specific application scenarios of big data technology in different stages of epidemic development, effectively responding to the disconnection between current technological development and realistic needs.

## 1. Introduction

Public health refers to “the science and practice of disease prevention and early diagnosis, control of infectious diseases, health education, injury prevention, sanitation, and protection against environmental hazards” (1). Modern public health was born in the 18th century, and with the increasing maturity of human knowledge and practice of public health, there is now a consensus on the definition of public health emergencies–events that have a serious impact on public health, including epidemic of major infectious diseases, animal epidemics, mass illness of unknown origin, grave food and occupational poisoning, etc. Public health emergencies are characterized by suddenness, complexity, severity, etc. (2–4). Since the 21st century, global public health emergencies have occurred frequently: the Severe Acute Respiratory Syndrome (SARS) outbreak in 2003 (5), Influenza A (H1N1) outbreak in 2009 (6), Middle East Respiratory Syndrome (MERS) outbreak in 2015 (7), and novel coronavirus (COVID-19) outbreak in 2019 (8). Frequent public health emergencies have caused significant damage to people's lives and health as well as the operation of the economy and society (9). Today, the world is still fighting against COVID-19, at a critical point of great change, differentiation, and adjustment. As of October 12, 2022, the total number of confirmed COVID-19 cases worldwide has reached 626,679,171 with 6,565,147 deaths (10). Faced with such a severe situation, China has adopted policies such as road closures, traffic restrictions, and strict home-staying to prevent the spread of the epidemic. Although these traditional manual approaches have been effective in controlling the epidemic, it has revealed many drawbacks that not only pose challenges to urban management, but also have far-reaching effects on the daily lives of urban residents (11, 12). Therefore, how to adopt new ideas, new technologies, and intelligent responses to improve the scientific nature of the work are top priorities of epidemic prevention and control.

Big Data refers to a huge data set that cannot be effectively processed in a short period of time, with large scale, wide dimensionality, and high scalability. First proposed by Alvin Toffler in 1980 in the book *The Third Wave*, it has 4V characteristics: volume, variety, velocity, and value (13). Big data technology and the governance of public health emergencies have significant applicability: from a policy standpoint, the Chinese state has introduced a series of policies related to strengthening the application of big data technology clusters in the field of public health, and big data has been implicitly reshaping and transforming the external ecosystem of the government. In June 2016, the *Guiding Opinions on Promoting and Regulating the Development of Big Data Application in Health Care* issued by the General Office of the State Council of the People's Republic of China, incorporated the development of big data applications in health care into the national big data strategic layout (14). In April 2018, the General Office of the State Council of the People's Republic of China issued the *Opinions on Promoting the Development of Internet Plus Medical Health* (15), encouraging medical and health institutions to cooperate with Internet companies to strengthen the intelligent monitoring of infectious diseases and other diseases. In February 2020, the Office of the Central Cyberspace Affairs Commission issued the *Notice on the Good Protection of Personal Information Using Big Data to Support Joint Prevention and Control Work* (16), encouraging capable enterprises to use big data to analyze and predict the flow of confirmed and suspected patients, their close contacts, and other highly-relevant populations. In terms of realistic needs, big data technology based on data and algorithms can incorporate the epidemic coverage and surrounding areas into the governance framework presented by data. With its powerful algorithmic logic and calculation rules, it can realize the accurate perception of the external environment to enhance risk diagnosis and early warning capability, which also determines the strong demand for big data technology in the management of public health emergencies (17).

To prevent and control global public health emergencies, most countries rely on institutional public health systems to provide early warning of infectious diseases. This traditional approach faces problems such as insufficient information and delayed response. With the advent of the information society, big data technology provides more diversified information sources and intelligent information processing methods for epidemic prevention and control, which shows great potential for development. The construction of a big data prevention and control model for public health emergencies is of great significance for promoting the deep integration of big data and public health, making up for the shortcomings of the disconnect between big data applications and actual needs, and improving the prevention and control capacity of public health emergencies. Therefore, based on Chinese experience of big data technology development and epidemic prevention and control, this paper conducts an exploratory study on the construction of a big data prevention and control model for public health emergencies using the qualitative research method of grounded theory, aiming to provide references for decision-making and improve the efficiencies of anti-epidemic campaigns. The contributions of this study include the following aspects: (1) The DSA model of big data prevention and control of public health emergencies is constructed in three dimensions: data layer, subject layer, and application layer, which actively responds to the new requirements of the top-level design of the national system in the field of public health. It is an important step to promote the deep integration of big data with public health and improve the technological innovation of epidemic prevention and control. (2) Unblocking channels of information collection, exchange, and transmission. The big data information in public health emergencies has been divided into four categories: “epidemic big data,” “healthcare big data,” “government open big data,” and “Internet big data.” It also analyzes the process of how data can realize added-value, and enhances the timeliness and integrity of public health-related information. (3) From a holistic perspective, the correlation and interaction of all necessary elements in epidemic prevention and control are comprehensively analyzed to achieve multi-source information processing and precise information positioning, complete the transformation from group management to individual tracking, and enhance the rigor and accuracy of epidemic prevention and control. (4) The specific application scenarios of big data technology in the incubation period, outbreak period, and recovery period of public health emergencies are analyzed, and macro policy advocacy is applied to epidemic prevention and control, which effectively solves the extreme lack of intrinsic correlation between “policy and practice.” It provides a different way of thinking about governance of public health emergencies compared to previous studies.

## 2. Literature review

The increasing perfection and timely application of big data technologies have changed the perception of information related to public health emergencies in the industry and academia, and have gradually exerted an important influence on the thinking paradigm and approach to crisis response. Experts and scholars at home and abroad have paid great attention to the application of big data in the management of public health emergencies, and a multidisciplinary research situation has been formed, with public health, emergency management, and intelligence science as the main disciplines. From the perspective of public health disciplines, it is mainly about the impact and challenges of big data in the medical field. For example, Pham et al. proposed combining SIR models with Machine Learning and Deep Learning models based on big data technologies that can send timely alert messages to governments and policymakers to act in advance of an outbreak (18). Lopez et al. effectively predicted the epidemiological impact of influenza in Vellore, India, by constructing a big data spatial model (19). Li et al. constructed a false epidemic information identification model to identify reliable information sources based on the analysis of health care big data, which divided the epidemic information screening and public opinion prevention and control research into two modules (20). Bragazzi et al. proposed that big data can help in handling the huge, unprecedented amount of data derived from public health surveillance, real-time epidemic outbreaks monitoring, trend now-casting/forecasting, regular situation briefing, and updating from governmental institutions and organisms (21). Liu et al. established a multi-stage time-delayed SEIR epidemic model based on the classic susceptible, exposed, infected, and recovered (SEIR) epidemic model and epidemic data in Guangzhou, which is suitable for epidemic research in Guangzhou and effectively solves the problem of optimizing the site selection decision for emergency medical facilities for public health emergencies in China (22). From the perspective of emergency management disciplines, the main research is about the mechanism and practice of the role of big data in public health emergencies. For example, Naudé proposed that big data has actual and potential uses in the fight against COVID-19 such as tracking and prediction, diagnosis and prognosis, treatment, and vaccine, which are used throughout the process of Reduction, Readiness, Response, and Recovery in the 4R crisis management theory, and that a careful balance between data privacy and public health needs to be maintained in the future (23). Lee et al. used the issue of mask-wearing as a research topic and applied big data analysis methods to international media reports on COVID-19 pandemic crisis themes conducted a thematic network analysis (24). Zhang H used big data technology to construct a multi-role portrait system model, which can provide data panorama support for epidemic monitoring, early warning, and investigation, and provide new ideas for the prevention and control of infectious diseases (25). From the perspective of intellectual discipline, it is mainly about the construction of intelligent systems and models for public health emergencies in the era of big data. For example, Mohamadou proposed that the intelligence model constructed by combining COVID-19 data such as “medical images, population movements, and case reports” with artificial intelligence can enhance the understanding of disease transmission and evaluation of preventive measures, so as to detect infected patients early and accurately (26). Esposito introduced and compared different data-driven epidemiological intelligence strategies (DDEIS) developed based on DPTT, and analyzed the extent to which DDEIS can effectively achieve the goal of quickly returning to normal cities and minimizing the risk of epidemic recurrence (27). Yin et al. fused the continuous mechanisms of Big Data Intelligent Innovation (BDII) into a complex network, and constructed a three-dimensional collaborative epidemic prevention model to reveal the effectiveness of continuous epidemic prevention under different big data intelligent emergency management policy levels (28).

According to the research results, scholars have made preliminary explorations on the integration of big data and the management of public health emergency governance, ranging from public health risk assessment to emergency capacity-building. However, there are still the following inadequacies: The existing research mainly focuses on the response to public health emergencies, lacking a comprehensive analysis of the interaction between the necessary elements in the emergency governance process from a holistic perspective, which has led to the insufficient application of big data in the management of public health emergencies. The intrinsic connection between macro policy advocacy and practical application is severed, making the deep integration of data, subjects, and applications virtually impossible. Specifically, at the beginning of COVID-19 outbreak, some medical institutions and enterprises in China tried to apply emerging technology with “big data” as the information chain in epidemic prevention and control, such as cloud computing, artificial intelligence, drones, and blockchain. These technologies have played an active role in online medical care, epidemic information processing, and precise management of the mobile population. However, in general, the application of big data technology in the front line of prevention and control is relatively limited at this stage, and the quality and efficiency of epidemic prevention and control work are low due to the lack of crucial technical support, resulting in a depression situation where hundreds of millions of people were home quarantine in China.

Therefore, compared with existing studies, this study aims to address the following questions: How to integrate and optimize the big data technologies currently applied to the prevention and control of public health emergencies, so that the fragmented practical experience can be upgraded into a systematic model, and then guide government departments to carry out accurate and intelligent prevention and control of epidemics? The research on this issue can be broken down into several smaller sub-questions: (1) How to integrate cross-industry, cross-region, and cross-domain epidemic big data information into one systemic framework to fully resolve the deficiencies in disjointed structures, management, and channels? (2) In epidemic prevention and control, what are the differences in demand for information and data among different subjects? How to promote the sharing of information resources and cooperative governance among multiple subjects to avoid the phenomenon of “information island”? (3) What are the practical applications of big data technology at different stages of the management of public health emergencies, from risk prevention, response and disposal to recovery and reconstruction? How to solve the problem that technological development is out of step with real needs?

## 3. Method and data

### 3.1. Methodology

The grounded theory originated in the field of sociology and is a scientific qualitative research method first proposed by sociologists Glaser and Strauss to address current scholars' doubts about positivism, and was initially applied to the treatment of dying patients by hospital medical staff. This method collects first-hand materials and data, abstracts and conceptualizes them, and then analyzes, compares, and summarizes them from top to bottom, so as to extract concepts and categories, and deduce and infer the research theory on this basis. In order to make the research theory more representative, the grounded theory research method requires the researcher to be highly sensitive to the data, and the data text must be comprehensive, authentic, and representative.

It is applicable and feasible to apply the grounded theory research method to the study of the construction of big data prevention and control model for public health emergencies. (1) Applicability, first of all, the research object of this paper is “prevention and control of public health emergencies,” because the development of epidemics is influenced by many factors from risk prevention, response and disposal to recovery and reconstruction, and the analysis of it needs to be based on the repeated comparison, verification, and generalization of a large amount of textual data. As a more advanced method in qualitative research, the grounded theory is suitable for this research. Secondly, the grounded theory provides a scientific research method to construct theories and discover mechanisms. The spread of public health emergencies is essentially a problem induced by the evolution of spatial and temporal processes, and in this evolutionary process, epidemic data are generally widely distributed and collected through scattered channels, etc. The ability of the grounded theory to explore hidden information and to conceptualize and analyze it is a great advantage in this research. (2) Feasibility, the research on the construction of big data prevention and control model for public health emergencies is rooted in realistic case texts and scientific theoretical achievements, which can effectively avoid the undesirable situation that theory is seriously detached from practice. In addition, the core of the grounded theory lies in the coding and deepening of the original data layer by layer, and it values the study of data. In today's era of big data of information explosion, government, medical institutions, enterprises, media, the public, and other subjects will report a lot of information on epidemic prevention and control, and the information needed for the study is abundant and easy to obtain, so it is feasible to use the grounded theory to study this topic.

**Figure 1:**
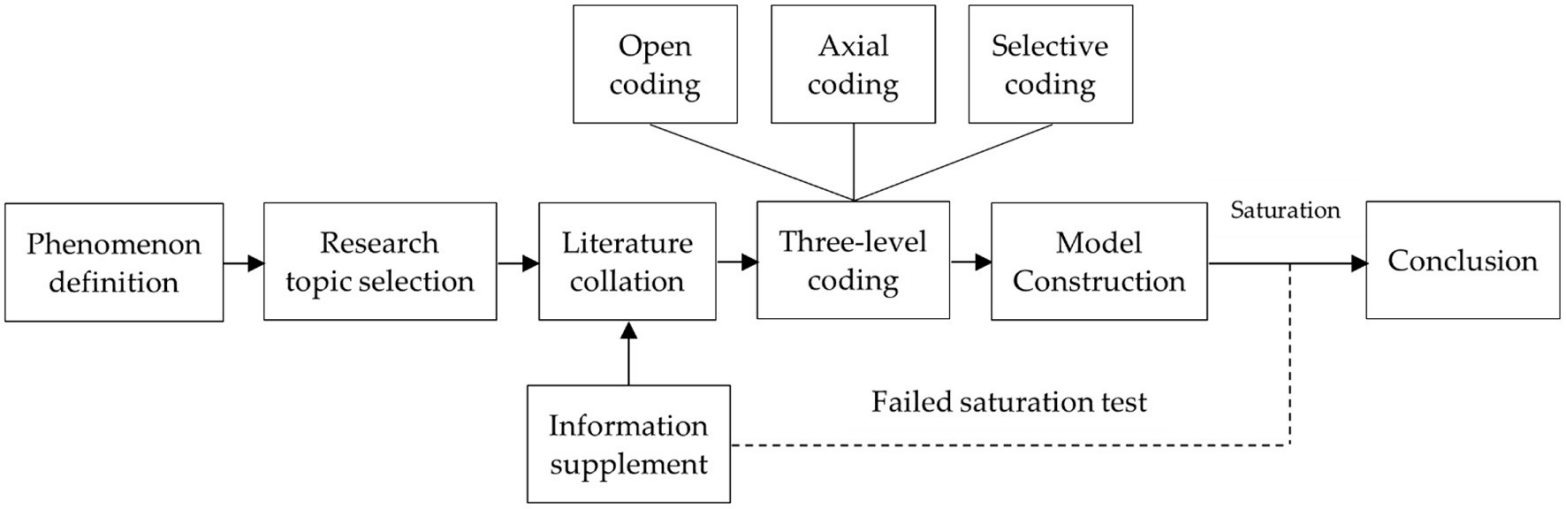
Research process based on grounded theory.

The research process based on the grounded theory includes the following steps (29, 30): (1) Determine the research topic, collect primary data purposefully through data survey and literature reading, and eliminate irrelevant data on the basis of ensuring the completeness and comprehensiveness of the data. (2) Data coding is performed on the primary data, concepts are generated from the data through coding, and relevant links between concepts are established to derive theories. The coding process includes open coding, axial coding, and selective coding. (3) Carry out a theoretical saturation test, and the conclusions are generally considered to be theoretically saturated if no new categories can be formed from the original data. (4) Build a theoretical model, and strive for a high degree of integration between the model and its application. This paper analyzes the data with the help of Nvivo 12 software in the process of data sorting and coding. The research process is shown in Figure 1.

### 3.2. Data sources

**Figure 2:**
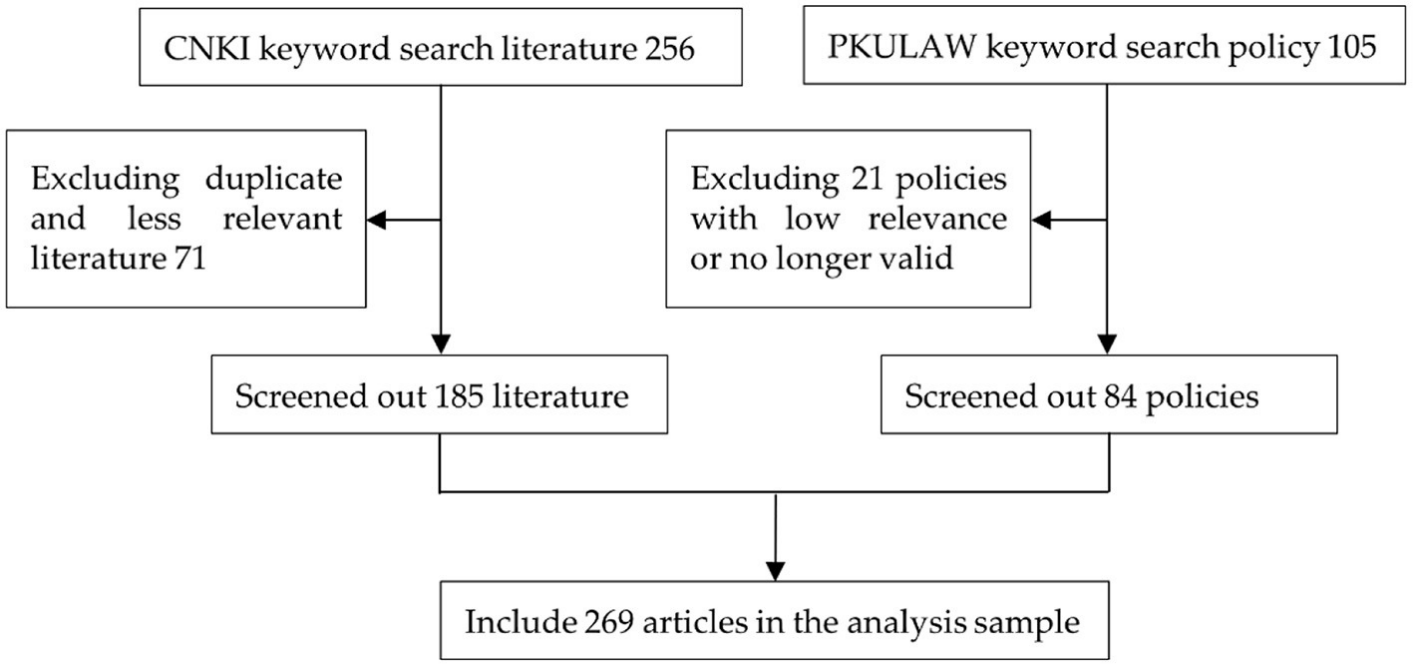
Literature and policy text rooting data sources.

Since literature reading is one of the important ways to source data in qualitative research, this paper used “public health,” “big data” and “epidemic prevention and control” as keywords to search relevant literature from CNKI, preliminarily selecting 256 papers. After reading and screening the papers one by one, 185 papers with strong relevance were selected as the analysis samples by eliminating the duplicate papers and those with low relevance. In addition, through various channels such as the Chinese General Legal Knowledge Resources Database, Chinese government websites, and the laws and regulations database of Peking University, “medical and health big data” “health science and technology innovation” “Internet medical” as keywords, the search time span was up to January 2022, and the search scope included various policies and measures, regulations, rules, laws and decrees promulgated by the central government (State Council, ministries and commissions) as well as local governments. Initially, 105 policies were selected, and 84 materials were finally screened out by eliminating those policies that were not highly relevant or had lapsed, as shown in Figure 2. The selection of the above textual data was based on the principles of openness, authority, and relevance to ensure the completeness and representativeness of the obtained data to the maximum extent.

## 4. Data coding and model construction

### 4.1. Open coding

Open coding is to gradually reduce the original data, get the initial concept and category, and determine the subordinate relationship between them, reflecting the connotation of the original data. In the open coding stage, the researchers are required to adhere to the principle of “believe everything and believe nothing,” abandon personal perception and habitual thinking, and ensure the objectivity of the coding results.

In the open coding process, the first step is data decoding: multiple researchers conduct tag construction (here labeled by TX) and concept extraction (here labeled by EX) word by word. If there are differences of opinion, discuss and reach a consensus to eliminate the influence of personal bias on the research results as much as possible. Secondly, the concept categories are summarized by comparing the initial concepts obtained until no new concepts are extracted. Finally, the conceptual categories reflecting the same phenomena are expressed in a more abstract set of concepts, i.e., the initial category (labeled here with IX) is formed. Following this process a total of 251 labels were given in this study, resulting in 53 concepts and finally 36 initial categories, which are coded as shown in Table 1.

**Table 1:** Open coding preliminary categorization.

**Tag construction**	**Concept extraction**	**Initial category**
T_1_ Data technology	E_1_ Epidemic type	I_1_ Government
T_2_ Data drive	E_2_ epidemic hazard	I_2_ Health care big data
T_3_ Data environment	E_3_ Epidemic scope	I_3_ Healthcare resource
T_4_ Public data	E_4_ Clinical treatment	I_4_ Path prediction
T_5_ Data openness	E_5_ Web crawler	I_5_ Data collection
T_6_ Data information	E_6_ Browse records	I_6_ Close contact screening
T... Data standards	E... Health record	I... Data analysis
Data classification	Community epidemic	Risk perception
Big data era	Population migration	Epidemic big data
Multi-source data	Vaccine development	Medical institutions
Data sharing	API interface	Epidemic monitoring
Data center	Vacancy filling	Media
Massive data	Standard treatment	Epidemic traceability
SEIR model	Epidemic evolution	Intelligent medical
Emergency response	Correlation analysis	Clinical data
Database building	Data interpretation	Government big data
Questionnaire system	CDC	Platform data set
Secondary disaster	Research institutes	Program optimization
Epidemiology	Sina Weibo	Information authenticity
Close coordination	Planning coordination	Helping to resume work
Regional distribution	Virus traceability	Visual display
Transmission trends	Isolation and treatment	Medical treatment
Viral variation	Behavioral motivation	Data collaboration
Ecology	Internet consultation	Public
Biogeography	Online drug purchase	Decision collaboration
Pathogens	Regional joint defense	Enterprises
Matrix	Negotiation decision	Epidemic warning
Rodents	Wrong decision	Goal coordination
Reverse tracing	Risk monitoring	Internet big data
Corporate interest	Misdiagnosis	Infected person tracing
Guided care	Treatment pressure	Data processing
Sterilization	Information sharing	Global needs
Relationship graph	Health education	Drug R&D
Education system	Big data profiling	Information timeliness
Place of visit inquiry	Mental health	Government interaction
Analysis report	Knowledge update	I_36_ Information source
Face recognition	Integrity analysis	
Infrared imaging	…	
Posture recognition	E_53_ Lessons learned	
Resumption of work		
…		
T_251_ Online office		

### 4.2. Axial coding

The process of 36 initial categories derived from open coding only ensures the objectivity of the data. The categories are relatively independent of each other and difficult to establish connections, so the second step is to perform axial coding. Spindle coding, also known as secondary coding, is a process of further classifying, condensing, and refining the initial categories obtained in open coding. Its main task is to find and establish the relationship between categories, so as to develop the main category and sub-category and complete the transition stage from empirical description to abstract analysis. In axial coding, it should not be limited to the concept category formed by open coding. It is equally important to keep the openness of data and thinking at this stage. Eight main categories are finally summarized through axial coding and coded as MX, which are subject classification, subject information demand, multi-subject collaboration, data type, data value-added, incubation period, outbreak period, and recovery period, as shown in Table 2.

**Table 2:** Axial coding forms the main category.

**Main category**	**Corresponding category**
M_1_ Subject classification	I_1_ Government; I_10_ Medical institutions; I_26_ Enterprises; I_12_ Media; I_24_ Public
M_2_ Subject information demand	I_32_ Global needs; I_15_ Clinical data; I_3_ Healthcare resource; I_17_ Platform data set; I_36_ Information source; I_19_ Information authenticity; I_34_ Information timeliness; I_8_ Risk perception; I_22_ Medical treatment
M_3_ Multi-subject collaboration	I_23_ Data collaboration; I_28_ Goal coordination; I_25_ Decision collaboration
M_4_ Data type	I_9_ Epidemic big data; I_2_ Health Care big data; I_16_ Government big data; I_29_ Internet big data
M_5_ Data value-added	I_5_ Data collection; I_31_ Data processing; I_7_ Data analysis; I_21_ Visual display
M_6_ Incubation period	I_11_ Epidemic monitoring; I_27_ Epidemic warning
M_7_ Outbreak period	I_13_ Epidemic traceability; I_4_ Path prediction; I_30_ Infected person tracing; I_6_ Close contact screening; I_14_ Intelligent medical; I_33_ Drug R&D
M_8_ Recovery period	I_20_ Helping to resume work; I_35_ Government interaction; I_18_ Program optimization

### 4.3. Selective coding and model construction

**Figure 3:**
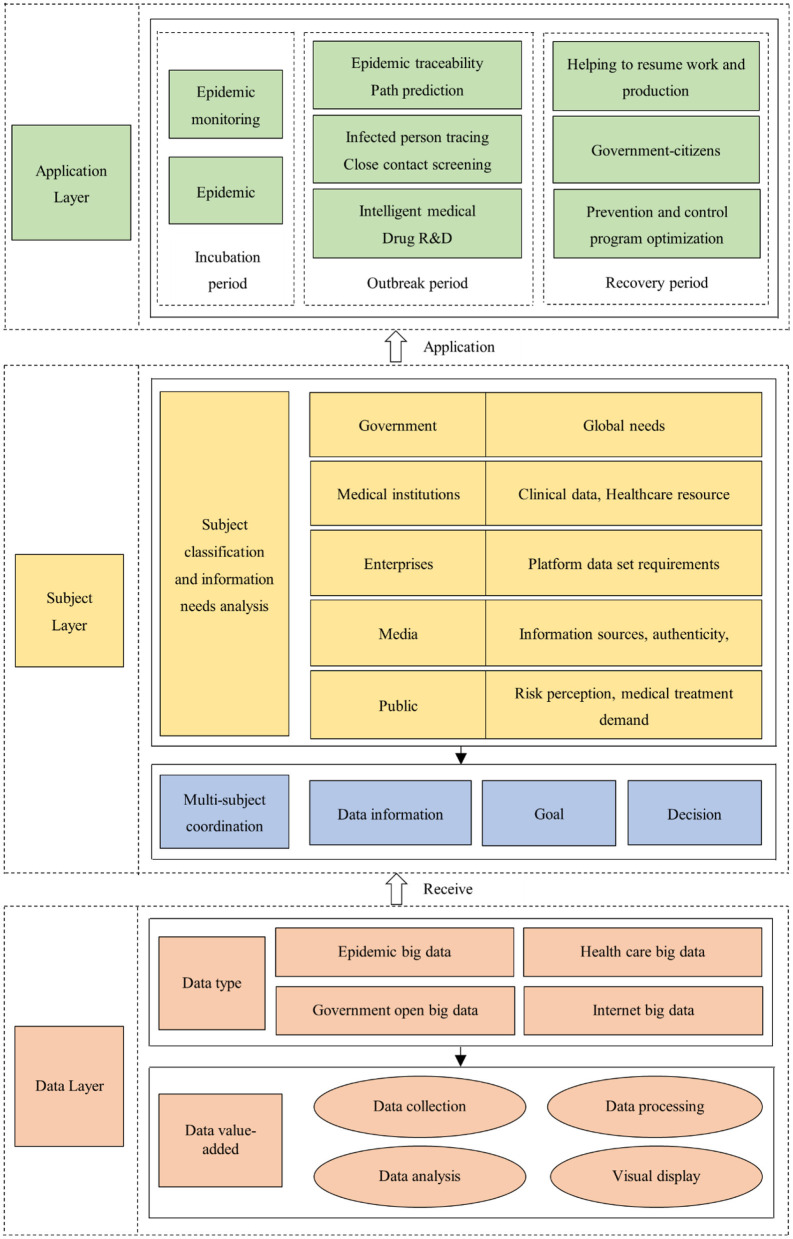
The “DSA” model of big data prevention and control of public health emergencies.

By clarifying the association paths between the main categories and the initial categories, the selective coding further abstracts and connotes the main category obtained in the secondary coding, so as to mine and form a leading core category, and finally build a grounded theoretical model that can cover all data. In the process of selecting the core categories, the following principles should be followed: (1) It should cover all phenomena and have more complete and subtle features compared with the main categories. (2) It should be selected based on the category with the highest frequency in the original data. (3) It has connections with all other categories and can be verified with data. (4) It can be developed to form a storyline, and its emergence and development are regular. Through continuous comparative research based on the above principles, the core categories are as follows: “data type” and “data value-added” are the underlying logic of the construction of big data prevention and control model for public health emergencies. These two categories are based on data, so they can be grouped into the core category of “data layer.” As the users of data, “subject classification,” “subject information demand” and “multi-subject collaboration” promote the digital prevention and control of epidemics, these three main categories can be refined into the core category of “subject layer;” The “incubation period,” “outbreak period,” and “recovery period” are the key stages of epidemic evolution, and the subjects must closely integrate with the application scenarios when using data, so these three main categories can be condensed into the core category of “application layer.” In summary, the core problem of this paper can be summarized as the event trail of the “data layer-subject layer-application layer” and formed into a theoretical model, as shown in Figure 3.

### 4.4. Saturation test

Theoretical saturation test refers to the process of stopping data collection and three-level coding of pre-reserved data to test whether the coding results obtained before are accurate when new concepts or categories cannot be found in the process of data analysis. The purpose of the saturation test includes the following two aspects: (1) To ensure the accuracy and credibility of the coding results and to eliminate the interference of human factors in coding to the maximum extent possible. (2) To ensure that the theoretical model obtained from the exploration has high validity. In this study, 50 research literature and policies were reserved as the data text for the saturation test (the 50 texts were randomly selected from the original data before the study started). Also, the procedure was the same as that for the theory construction in the paper–open coding, axial coding, and selective coding were performed on the text. The final 45 initial concepts can be obtained from the 53 previously constructed concepts, and no new concepts are found; After categorizing the 45 initial concepts, 35 initial categories are obtained. Except that the category of “prevention and control scheme optimization” does not appear, other categories can be found in the previous 36 categories; After axial coding of 45 initial categories, 8 main categories were obtained, and no new categories were mined, and the core categories obtained in the next step were the same. Therefore, it can be determined that the “big data prevention and control model for public health emergencies” constructed in this paper is theoretically saturated.

## 5. Interpretation of model content

Through the modeling and saturation test of the grounded theory, this study found that the three elements of “data-subject-application” play a prominent role in the digital prevention and control practice of epidemics, and constitute the basic framework of the model, so the model is referred to as the “DSA” model of big data prevention and control of public health emergencies. In order to make the results of model construction clearer and more comprehensive, the model is specifically explained as follows.

### 5.1. The data layer

This part mainly includes four aspects of data collection, processing, analysis, and visualization. The main task is to realize the value-added process of data–by carrying out a series of tasks on multi-source big data information closely related to the epidemic, fully extracting valuable information, opening up cross-border information integration channels, realizing the circulation of information among multiple subjects and preparing for the effective control and prevention of the epidemic (31).

From the perspective of data sources, some data such as social media, drug sales, search engines, electricity consumption, etc. do not appear to contain too much information related to the epidemic, and cannot replace clinical data as the mainstream. However, if these structured and unstructured data are combined through correlation analysis and refined using tools, it will be found that they not only have advantages in terms of data availability, but also reflect a picture that is more referential and valuable than clinical data. Referring to the views on data types put forward by scholars such as Fang (32), Liu (33), Wu (34), and Costa (35), this paper divides the data sources involved in public health emergencies into the following categories. First, the epidemic big data, such as the type, scope, level, hazard, etc. of infectious diseases. Second, the medical and healthcare big data, mainly including clinical treatment, pharmaceutical research and development, personal electronic health records, etc. Third, the government's open and public big data, mainly including population migration, community, traffic, environment, etc. Fourth, the internet big data, generally coming from public platforms such as social media, in addition to some information provided by the active participation of users, web browsing records, travel data, transaction data, etc. These data generated by individuals when using mobile phones may also become “passive” collection sources.

Data is usually collected through a combination of big data technology and traditional manual methods. With the main method of big data such as docking API interface, web crawler, and direct network transmission, the data from multiple departments such as municipal, health, telecommunication, and transportation are unified into a regional epidemic prevention and control analysis platform to realize real-time data collection and access, enrich data samples and improve collection efficiency. It is supplemented by traditional manual collection, which effectively avoids the problem of data limitations caused by too many blind spots in the network, and minimizes the “pseudo-big data phenomenon” caused by the “observer effect” and its negative effects using rational thinking and judgment of human beings.

Data processing is the process of improving data quality using methods such as cleaning, filling in vacancy values, and standardization. The most intuitive change brought by the convergence of data from multiple sectors is the expansion of data capacity and diversification of structure, which brings serious challenges to the data processing work. Therefore, the data format must be unified to achieve the integration of heterogeneous epidemic data from multiple sources. Natural language processing, semantic association, and other technologies can be fully used to automatically check and verify logical errors, content fragmentation, duplicate reporting and other issues to ensure data availability.

Data analysis is the core of the whole big data processing process, mainly including epidemic information relevance analysis, subject sentiment tendency analysis, and epidemic evolution prediction, etc. It works by converting multi-source data into mathematical models according to industry logic for business requirements, so that the machine can automatically output the results according to the algorithm requirements to meet decision-making needs (36, 37). Accordingly, data modeling to support business decision-making is the essence of data analytics, which also determines that the way of thinking must be transformed from causal analysis to knowledge discovery, from logical reasoning to association rulemaking, and from empirical judgment to deep processing of model fitting.

The visual display is the final result based on a comprehensive grasp of epidemic big data information, the core of which is to enhance data interpretation capabilities and avoid misleading people. It is generally presented visually in the form of charts and graphs to strengthen the effect of information reporting to relevant decision-makers so that they can understand the real-time development of the epidemic situation in a clear, accurate, and timely manner. For example, the heat map is an intuitive presentation based on visualization technology, which integrates information on geographical location, population flow, and group behavior of public health emergencies. The heat map significantly reduces the cognitive threshold of complex data for decision-makers so that they can participate in epidemic prevention and control work to the greatest extent.

Through the above analysis of the value-added process of big data in the management of public health emergencies, it can be found that its essence is to “extend” the tools of human cognition and exploration of the unknown with the support of data and algorithms, and gradually change people's working methods while solving the epidemic prevention and control problems. The collection, processing, and analysis of information by big data is a necessary path of technology development, and data will certainly play an important role as “blood” in the actual epidemic prevention and control applications. This means that to enjoy intelligent and informative services, people must transfer their information as a prerequisite. Therefore, the use of big data technology to help prevent and control epidemics while taking into account data security and personal privacy protection, so as to give full play to the real value and positive effects of big data technology, should become a key issue for research.

### 5.2. The subject layer

The main task of this part is to promote consensus among multiple subjects on collaborative management of epidemics based on the access to multi-source big data information by epidemic prevention and control subjects according to their needs, and realize all-round and seamless governance through communication, cooperation, and grid-based integration.

#### 5.2.1. Subject classification and their needs analysis

Participants in epidemic management mainly include the government, medical institutions, enterprises, media, and the public. Participants' needs in the epidemic response process can both reflect the selection preferences of the public and drive the steady improvement of the epidemic prevention and control work. The government assumes the overall planning and coordination function in response to public health emergencies, and its main task is to effectively integrate the interests and resources of all parties and establish a cooperative and complementary relationship among all subjects, forming a compound governance model of risk-sharing and coexistence under government leadership. On the one hand, we need to enable accurate prevention of emergencies and send out monitoring and warning signals. On the other hand, it needs to respond quickly to effectively control the spread of the epidemic. In addition, it has the unshirkable responsibility for the effective allocation of resources and the recovery and reconstruction after the epidemic. Therefore, in epidemic prevention and control, the government's demand for data and information is global, and runs through the incubation, outbreak, and recovery periods of the epidemic.

Medical institutions, including hospitals, CDCs, medical research institutes, etc. are mainly responsible of the isolation and treatment of patients, virus origin-tracing, transmission cause analysis, and diagnosis plan selection, which are of great significance to epidemic early warning, situation assessment, and crisis decision-making. The needs of medical institutions for data and information include medical network resources, epidemic prevention observation data, patient clinical data, disease diagnosis case base, etc. For example, according to the data of suspected and confirmed cases in a certain area, the demand for medical supplies can be accurately grasped. Combined with the growth rate of confirmed patients and epidemiological regularity, the scale of the potentially infected population can be described to determine the emergency response level of the epidemic.

Enterprises including Alibaba, Sina, Baidu, etc., can obtain abundant Internet data based on huge user groups. For example, Baidu Maps can accurately grasp the migration status of population flow through personal positioning. Sina Weibo can timely send epidemic warning signals through the frequency of user searches and information interaction feedback. It can be seen that the demand of enterprises for data information is “a platform data set that can fully reflect public opinion and needs based on public behavior and motivation.” Companies track and analyze the real-time data obtained from the platform, and then provide society with products and services that are more targeted, more practical, and more satisfying to the needs of the public.

Media are the mouthpiece of government departments. Their objective, accurate and timely reporting of epidemic information can effectively satisfy the public's extreme desire for information due to panic and blind obedience, and nip negative public opinion in the bud or reduce its negative impact. The media's demands for data and information are mainly manifested in the following aspects. First, is the need for information sources, as the “gatekeeper” for selective dissemination of events, the media has a strong guiding power over the immediate reaction of the public to receive information, and it is crucial for the media to grasp first-hand information on time. Second, the demand for information authenticity. Revealing the truth, reflecting the reality of the problem, and correctly guiding public opinion are the responsibilities of the media, which requires the media to have a precise command of events in terms of authenticity. Third, the demand for timeliness of the information. The delay of information disclosure or information disclosure without a considered order will make it difficult for the public to distinguish the authenticity of the information, making the situation worse.

The public is the smallest unit in response to public health emergencies, and also the “basic statistical population” of most of the epidemic big data information, such as communication data, consumer behavior data, traffic data, etc. The behaviors of each member of the public have a significant impact on epidemic prevention and control. The public's demand for data information mainly includes two aspects. Risk perception is most important the public needs to know information about the spread of the epidemic, patient treatment and severity, and then perceive the risk of public health emergencies to take appropriate preventive measures. The second is the demand for information on medical treatment and materials, including hospitals designated for medical treatment, online consultation, contactless drug purchase, etc.

#### 5.2.2. Coordination and cooperation of multiple subjects

To effectively respond to COVID-19, China has established a joint prevention and control mechanism under the State Council, which is vertically classified into three levels of national, provincial, and local, and sets up a horizontal organization structure, including working groups for medical treatment, scientific research, epidemic prevention and control, logistics material support, and publicity. The work was both relatively independent and managed centrally to form an effective synergy force for epidemic prevention and control (38). The mechanism emphasizes three synergies between organizations:

1. Data and information synergy. The information sources and analysis of a single subject often have great limitations, so an information platform for public health emergencies should be built to share epidemic information and break the barrier of information sharing to the maximum.

2. Goal coordination. The division of professional functions of different subjects determines the difference in their goals, and the divergence of goals triggers behavioral conflicts and buck-passing. Therefore, goal consistency is the core element for multiple subjects participating in epidemic prevention and control.

3. Decision-making coordination (39). Decision-making coordination is a “consultative” decision-making process that requires coordination, communication, negotiation, and compromise among multiple organizations. In this way, arbitrary could be avoided, and problems of centralized power and conflicting functions could be alleviated.

### 5.3. The application layer

Big data must be combined with specific application scenarios to realize its full value (40). Based on various models of epidemic evolution at home and abroad, this paper divides the evolution of public health emergencies into three stages: the incubation period, the outbreak period, and the recovery period. The following analyzes the main application scenarios of big data in the three stages of epidemic development to fully demonstrate the specific role of big data.

#### 5.3.1. The incubation period

This phase focuses on epidemic awareness, both monitoring and early warning–through the analysis of epidemic characteristics, information needs of multiple subjects targeted collection of various types of epidemic data, the use of multi-source information to do the corresponding monitoring of the global situation. When the monitoring results exceed the preset threshold, early warning signals shall be sent to relevant departments and the public in time, to realize “prevention first.”

##### 5.3.1.1. Epidemic surveillance based on internet big data

Internet big data refers to the traces left by individuals when using the Internet. Internet big data related to public health mainly includes information shared on social networking platforms, information recorded by search engines, and information reported by news media. The reason why these multiple sources of information can be used for epidemic risk monitoring is based on a common premise: not all patients will go to hospitals for checkups when infectious diseases are prevalent, and some of them will look up information through the Internet to seek help. Therefore, one may capture and track these data (e.g., keyword frequency statistics) to predict disease incidence. For example, foreign social platforms such as Twitter and Facebook, as well as domestic Weibo and WeChat can analyze the spatial clusters of health and disease-related content and use this “aggregated intelligence” to assist in epidemic monitoring; In 2009, the Google search engine successfully predicted the outbreak of influenza by analyzing the total search volume and frequency of keywords in a specific region, and its influenza information reports were released more than a week earlier than the Centers for Disease Control (CDC) in the United States (41). The Global Public Health Intelligence Network (GPHIN), established by the World Health Organization, links more than 3,000 news sources in nine languages around the world in an aggregator to identify public health threats in a timely manner through real-time translation and data processing (42).

##### 5.3.1.2. Epidemic surveillance based on medical and health big data

The monitoring of epidemics by medical and health big data is mainly realized through citizen behavior and symptom monitoring, among which drug retail data and health electronic records data provide the main support for epidemic monitoring. Drug retail data (respiratory and intestinal drugs, antiviral drugs, cold drugs, etc.) can reflect the occurrence of diseases to a certain extent, and have significant advantages in terms of geographical location identification and early warning timeliness. It has been shown that sales of antiviral drugs are closely related to the total number of confirmed cases of influenza, and sales of thermometers and hand sanitizers are also significantly associated with influenza cases (43). Based on this, the UK has begun to use OTC sales records for monitoring the spatial and temporal patterns of influenza activity. The convergence of data on cases, clinical diagnoses, and treatment outcomes recorded in electronic health records can effectively help build epidemiological analysis models, detect abnormal aggregations of infectious diseases in time and space, and improve the sensitivity to new diseases and explosive epidemic situations.

##### 5.3.1.3. Precise information pushing early warning based on big data technology

The early warning of public health emergencies is based on monitoring. When the monitoring results exceed the preset threshold, it is crucial to deliver the early warning information to the relevant emergency management departments and the public in time (44). Unlike ordinary emergencies, public health events have group differences in the immunity of the public to viruses due to the biological characteristics of viruses (e.g., the elderly, the weak, the sick, the disabled, the young, and women, etc.), which puts forward new requirements for the accuracy of information delivery. The information pushes warnings based on big data following the thinking of “let the information find the user” and has the characteristics of “target user accuracy,” “user acquisition timeliness,” “information and user demand consistency,” etc. It relies on user identification, automatic recording, information retrieval, and other technologies to understand different users' immediate and potential needs, and realizes personalized information delivery through the Internet, social networking platforms, broadcasting, mobile communication, and other channels. For example, with the help of Internet social platforms such as Tencent and Sina, information containing early warning signals is broadcasted on a rolling basis in the user interface of specific regions to achieve zero error and full coverage of early warning.

#### 5.3.2. The outbreak period

The focus of this phase is on epidemic response, collecting and analyzing epidemic-related information, and assessing the type, level, region, and possible hazards of the outbreak, while tracking the epidemic process and intervening in a timely manner to reduce negative impacts and losses.

##### 5.3.2.1. Epidemic traceability and transmission path prediction based on big data

By deeply integrating medical data, Internet data, and open government data, big data technology can realize the interconnection between social reality and epidemic development, which helps trace the epidemic's source and predict the transmission path. Based on daily case statistics, statistical models can be built with the help of machine learning and correlation analysis technologies on medical admissions, regional distribution, regulatory isolation, etc. The models can not only retrace the trend of the spread of the epidemic to locate its source, but also provide an important scientific basis for predicting the spreading path of the epidemic. For example, the European CDC and the Umea University in Sweden used data such as Twitter, climate change, the estimated number of Aedes albopictus mosquitoes, and international air passenger traffic to build a big data model to successfully project the origin and spreading path of the 2017 Chikungunya virus outbreak in Europe (45). Xumao Zhao and other scholars from Lanzhou University successfully used big data to retrace the trend of virus spread at the early stage of the outbreak of COVID-19. They concluded that “the population exported from Wuhan is the main threat to the spread of COVID-19 in China” (46).

##### 5.3.2.2. Infected person tracking and close contact screening based on big data

Accurate identification and timely isolation of infected persons and close contacts is a key link to contain the further spread of the epidemic. Traditionally, manual investigation (household interviews, questionnaires, etc.) is mainly used to accomplish this, which has shortcomings in terms of cost, efficiency and accuracy, and may miss the best time for epidemic prevention and control. The ubiquitous network and intelligent sensing technology based on big data can track population flow, individual relationship mapping, facility environment, etc. in real-time, and accurately locate infected persons and close contacts. Through the comprehensive application of temperature-sensing camera and public security face database, combined with the recognition of physical features by multi-modal algorithm, body temperature data collection can be completed within 2 m and matched with identity information quickly. Once a fever patient is found, an alarm signal will be issued automatically to realize the “carpet” investigation of suspected cases. In addition, with the help of telecommunications big data, migration big data, e-billing and other information, correlation analysis and social network distance measurement can provide users with “14 days to visit the place of inquiry” service, so as to quickly screen close contacts and take isolation and treatment measures. For example, the “green, yellow, and red” three-color health code, which is widely used during the prevention and control of COVID-19, is generated by using big data to quantify the three dimensions of time, space, and interpersonal relationships.

##### 5.3.2.3. Intelligent medical and drug R&D based on big data

Currently, the most typical application in the field of intelligent medical applications empowered by big data is none other than medical image analysis (13, 47). The diagnosis of COVID-19 mainly relies on chest CT images and nucleic acid detection. However, the traditional manual reading mode is far from satisfying the consultation needs of many patients, which leads to misdiagnosis and omission and is also challenged by insufficient medical staff. The wide application of image recognition technology based on convolutional neural networks in the medical field has effectively solved this problem. The technology is based on big data deep learning algorithms, which automatically construct pattern recognition by repeatedly training the machine with medical images, greatly reducing the pressure on the medical staff. For example, the COVID-19 AI-assisted diagnosis system created by Alibaba Cloud Computing can handle a case in 20 s on average, which is 50 times faster than doctors. In drug research and development, the new drugs go through a series of processes such as drug screening, pharmacological analysis, and safety testing, which require extremely high economic and time costs, and the use of big data-based evaluation networks and Monte Carlo Tree Search algorithms (MCTS) (48) can effectively alleviate this problem by selecting the safest from tens of thousands of compounds as the best alternative for new drugs.

#### 5.3.3. The recovery period

In the recovery stage, the epidemic is effectively controlled, and the affected areas and people's lives begin to recover orderly. The focus of this phase is to sort out, evaluate, and analyze the information on the whole process of the epidemic, propose recovery and reconstruction plans, and at the same time summarize the experience of epidemic prevention and control to provide theoretical guidance for future related work.

##### 5.3.3.1. Big data helps enterprises to resume work and production

Big data can guide relevant departments to resume production and economic restart plans. On the one hand, through the construction of algorithmic models and data-sharing platforms, it can make forward-looking predictions on the trend of the epidemic and distribution of high-risk areas, and determine whether a series of emergency response measures for the epidemic can be lifted, so as to provide scientific support for relevant decision-making. For example, the big data platform of “Resumption of Work and Production Analysis” built by the Tianjin Municipal Tax Service has established a real-time data sharing mechanism with 18 departments, including the Municipal Government, the Municipal Finance Bureau, the Municipal Bureau Statistics, the Municipal Commission of Housing and Urban-Rural Development, and the Administration for Market Regulation, and has shared up to 6 million pieces of data, which provides strong support for the work according to local conditions (49). On the other hand, the full exploration and application of big data on electricity can assist the government in applying early intervention and dynamic management to the company that resumes work and production. Taking the average daily power consumption of enterprises during the outbreak of the epidemic as the basic data and the fluctuation of the average daily electricity consumption as the focus point (focusing on enterprises with electricity consumption growth rate above 100%), combined with the model calculation, the “big data portrait” of enterprises in the city can be constructed to realize the accurate control of the dynamics of enterprises' resumption of work and production.

##### 5.3.3.2. Big data promotes government-public interaction and epidemic prevention and control program optimization

In the recovery phase of the epidemic, the public is easily misled by inaccurate statements, which may induce secondary and derivative hazards of the epidemic, so it is essential to strengthen the construction of public mental health. Big data can analyze the behavior of netizens and information dissemination rules, and carry out intelligent government-public interaction based on a full understanding of the real needs of the public, to deliver timely and accurate information about the disposal of the epidemic and follow-up plans to the public, and nip the negative public opinion caused by rumors in the cradle. In addition, the virtual simulation technology based on big data can digitally reproduce the whole picture of the development of the epidemic, help the relevant departments to make systematic summaries of the causes, losses, and lessons learned from the outbreak, and realize the optimization of the epidemic prevention and control plan and the update of the historical case knowledge base.

## 6. Discussion

The essence of the “DSA” model for big data prevention and control of public health emergencies is a comprehensive body integrating “data acquisition and analysis,” “subject demand analysis and collaboration,” and “phased application of big data” with the goal of realizing intelligent, collaborative, and efficient epidemic control. Undeniably, there may be some shortcomings in this study. First, this paper is limited to a theoretical discussion. Collecting real data from public health emergencies, verifying, and improving the model will be the main work in the next stage. Second, this study attempts to explore and construct the “DSA” model from the data-subject-application multiple perspectives. However, there are many factors affecting the management of public health emergencies, such as national policy guidance, regional differences in epidemic prevention and control, virus types and transmission capacity, etc., all of which point out the direction of efforts for future work. Third, the selection of data samples in the construction of the DSA model is based on the policy texts and academic studies issued in China, which inevitably brings the question of whether the model is universal and representative in the international arena. Therefore, how to use big data technology to help prevent and control epidemics while taking into account the basic conditions of most countries in the international community is also the focus of the next research.

In addition, future research will focus on preventing “data leakage and privacy violation,” because the construction of the “DSA” model relies on big data technology, whether it is high precision mining, full sample collection, or high rule association and full data analysis, it cannot escape the fetters of “privacy protection” and “analysis bias.” For example, while enterprises use big data technology to provide intelligent services for epidemic prevention and control, it also poses a huge challenge to the protection of personal privacy: by collecting and linking the data footprints left by people on the Internet into a complete information chain, it is possible to draw some conclusions related to personal privacy, which can be used by criminals to commit fraud and bring economic losses or mental distress to individuals. In order to effectively respond to the challenges facing personal information protection, China has successively introduced laws and regulations such as the *Decision on Strengthening the Protection of Network Information*, the *Cybersecurity Law of the People's Republic of China*, and the *Civil Code of the People's Republic of China*, which clearly stipulate the basic principles of personal information processing activities and significantly enhance the protection of personal information. For example, there is no problem with collecting personal information on the whereabouts of individuals by scanning the “health code” in the context of epidemic prevention and control, but if the information is used to obtain further information on personal consumption preferences, home addresses or even resold to others, it involves the excessive collection and illegal leakage. Therefore, although we expect the full use of big data to bring its value into play, we cannot rely on it entirely. We must keep a clear head and think rationally in the wave of big data, use it as an effective tool rather than the ultimate means, and continue to promote fundamental changes in the governance mode of public health emergencies by increasing the depth of data and information intervention.

## 7. Conclusions

Public health emergencies are usually complex and long-term. The traditional response and disposal mode has been difficult to meet the practical needs of the prevention and control of various new infectious diseases. There is an urgent need to introduce the emerging technology group represented by big data, to achieve significant innovation in the concept, thinking, and technology related to prevention and control. Compared with existing research, the strength of this study lies in the construction of a generic model of big data prevention and control based on a holistic perspective and applicable to different stages and spatial scales. Based on the collection of real-time data, the model processes and obtains accurate information on the risk of public health emergencies to assist government departments in prediction and early warning, emergency response and control, decision-making and implementation, and recovery and reconstruction activities, to achieve effective control of public health emergencies. From the perspective of structure, the model includes three parts: data information source, subject's demand for data, and data application, and has the characteristics of real-time, accuracy, and purposefulness.

This paper conducts an exploratory study on the construction of a big data prevention and control model for public health emergencies by using grounded theory, a qualitative research method, with literature, policies, and regulations as research samples, and makes a grounded analysis through three-level coding and saturation test. The main results are as follows:

(1) This paper constructs a “DSA” model for the prevention and control of public health emergencies from the data layer, subject layer, and application layer to comprehensively reflect the interaction of different elements in the epidemic, to assist the government in making appropriate decisions and policy adjustments, integrating prevention and control measures, and promoting rapid economic growth and recovery.

(2) The “data layer” module of the DSA model integrates the automated collection, standardized processing, intelligent analysis, and visualization of data from multiple sources to build a basic data warehouse, integrating cross-industry, cross-region, and cross-domain information of the epidemic into one systematic framework, which facilitates information collection, communication, and transmission channels and enhances the timeliness and integrity of public health information.

(3) The “subject layer” module of the DSA model provides a comprehensive analysis of the differences in information needs of multiple subjects such as government, medical institutions, enterprises, media, and the public during the epidemic outbreak. The government's demand for data and information is global. The needs of medical institutions include medical network resources, epidemic prevention observation data, patient clinical data, and disease diagnosis case base. The needs of enterprises are “a platform data set that can fully reflect public opinion and needs based on public behavior and motivation.” The needs of the media include information sources, information authenticity, and information timeliness. The needs of the public include risk perception and medical treatment.

(4) The “application layer” module of the DSA model analyzes the application scenarios of big data technology in different stages of the “incubation period, outbreak period, and recovery period” of the epidemic development. In the incubation period, the main applications are epidemic monitoring and accurate information delivery. In the outbreak period, the main applications are epidemic tracing and transmission path prediction, infected person tracking and close contact screening, and intelligent medical and drug R&D. In the recovery period, the main applications are helping enterprises to resume work and production, promoting government-public interaction, and epidemic prevention and control plan optimization. These applications put macro policy initiatives into practice and contribute to the prevention and control of epidemics, effectively responding to the disconnection between current technological development and real demand.

## Data availability statement

The original contributions presented in the study are included in the article/supplementary material, further inquiries can be directed to the corresponding author.

## Author contributions

Conceptualization and writing—original draft preparation: HW and HY. Methodology: LL. Data curation: HY and LL. Writing—review and editing: HW, HY, and LL. All authors have read and agreed to the published version of the manuscript.
